# Toll-like receptor 4 (TLR4) is the major pattern recognition receptor triggering the protective effect of a *Candida albicans* extracellular vesicle-based vaccine prototype in murine systemic candidiasis

**DOI:** 10.1128/msphere.00467-24

**Published:** 2024-07-22

**Authors:** Leandro Honorato, Jhon J. Artunduaga Bonilla, Alessandro F. Valdez, Susana Frases, Glauber Ribeiro de Sousa Araújo, Albaniza Liuane Ribeiro do Nascimento Sabino, Natalia Martins da Silva, Larissa Ribeiro, Marina da Silva Ferreira, Julio Kornetz, Marcio L. Rodrigues, Iain Cunningham, Neil A. R. Gow, Attila Gacser, Allan J. Guimarães, Fabianno F. Dutra, Leonardo Nimrichter

**Affiliations:** 1 Laboratório de Glicobiologia de Eucariotos, Departamento de Microbiologia Geral, Instituto de Microbiologia, Universidade Federal do Rio de Janeiro, Rio de Janeiro, Brazil; 2 Laboratório de Biofísica de Fungos, Instituto de Biofísica Carlos Chagas Filhos (IBCCF), Universidade Federal do Rio de Janeiro, Rio de Janeiro, Brazil; 3 Rede Micologia, RJ, FAPERJ, Rio de Janeiro, Brazil; 4 Laboratório de Bioquímica e Imunologia das Micoses, Departamento de Microbiologia e Parasitologia, Instituto Biomédico, Universidade Federal Fluminense, Niterói, Rio de Janeiro, Brazil; 5 Instituto de Microbiologia Paulo de Góes, Universidade Federal do Rio de Janeiro, Rio de Janeiro, Brazil; 6 Instituto Carlos Chagas (ICC), Fundação Oswaldo Cruz (FIOCRUZ), Curitiba, Brazil; 7 Institute of Medical Sciences, University of Aberdeen, Aberdeen, United Kingdom; 8 MRC Centre for Medical Mycology, University of Exeter, Exeter, United Kingdom; 9 HCEMM-USZ Fungal Pathogens Research Group, Department of Microbiology, Faculty of Science and Informatics, University of Szeged, Szeged, Hungary; 10 Laboratório de Inflamação e Imunidade, Departamento de Imunologia, Instituto de Microbiologia, Universidade Federal do Rio de Janeiro, Rio de Janeiro, Brazil; University of Georgia, Athens, Georgia, USA

**Keywords:** *Candida albicans*, extracellular vesicles, vaccines

## Abstract

Systemic candidiasis remains a significant public health concern worldwide, with high mortality rates despite available antifungal drugs. Drug-resistant strains add to the urgency for alternative therapies. In this context, vaccination has reemerged as a prominent immune-based strategy. Extracellular vesicles (EVs), nanosized lipid bilayer particles, carry a diverse array of native fungal antigens, including proteins, nucleic acids, lipids, and glycans. Previous studies from our laboratory demonstrated that *Candida albicans* EVs triggered the innate immune response, activating bone marrow-derived dendritic cells (BMDCs) and potentially acting as a bridge between innate and adaptive immunity. Vaccination with *C. albicans* EVs induced the production of specific antibodies, modulated cytokine production, and provided protection in immunosuppressed mice infected with lethal *C. albicans* inoculum. To elucidate the mechanisms underlying EV-induced immune activation, our study investigated pathogen-associated molecular patterns (PAMPs) and pattern recognition receptors (PRRs) involved in EVs-phagocyte engagement. EVs from wild-type and mutant *C. albicans* strains with truncated mannoproteins were compared for their ability to stimulate BMDCs. Our findings revealed that EV decoration with *O*- and *N*-linked mannans and the presence of *β*-1,3-glucans and chitin oligomers may modulate the activation of specific PRRs, in particular Toll-like receptor 4 (TLR4) and dectin-1. The protective effect of vaccination with wild-type EVs was found to be dependent on TLR4. These results suggest that fungal EVs can be harnessed in vaccine formulations to selectively activate PRRs in phagocytes, offering potential avenues for combating or preventing candidiasis.

**IMPORTANCE:**

Systemic candidiasis is a serious global health concern with high mortality rates and growing drug resistance. Vaccination offers a promising solution. A unique approach involves using tiny lipid-coated particles called extracellular vesicles (EVs), which carry various fungal components. Previous studies found that *Candida albicans* EVs activate the immune response and may bridge the gap between innate and adaptive immunity. To understand this better, we investigated how these EVs activate immune cells. We demonstrated that specific components on EV surfaces, such as mannans and glucans, interact with receptors on immune cells, including Toll-like receptor 4 (TLR4) and dectin-1. Moreover, vaccinating with these EVs led to strong immune responses and full protection in mice infected with *Candida*. This work shows how harnessing fungal EVs might lead to effective vaccines against candidiasis.

## INTRODUCTION

Candidiasis impacts the quality of life of patients and the public health costs worldwide (1–4). Some *Candida* species are commensal, present in up to 60% of healthy individuals (2). Upon epithelial barrier breaches, dysbiosis, or immune dysregulation, *Candida* species can modulate and escape the host immunity, becoming a lethal pathogen (2, 5). Among all *Candida* species, *Candida albicans* is the most prevalent agent causing candidiasis (6–10).

Despite the current armamentarium of antifungal drugs, systemic candidiasis has a mortality rate of approximately 40% (4), but can reach up to 70% in South America (11). Furthermore, several strains resistant to azoles and echinocandins, usually the first choices for candidiasis treatment, have been documented (12, 13). Although potential new drugs are under investigation (14), there is an increasing interest in developing safe and efficient immune-based treatments (15, 16). Monovalent vaccine formulations have been successfully used in murine models of candidiasis (recently reviewed by Sahu and colleagues [16]). However, only two have reached human clinical trials (17), but so far none of them were licensed. PEV-7 and NDV-3/NDV-3A, carrying sequences for secreted-aspartyl protease-2 (SAP2) and the cell wall protein agglutinin-like sequence-3 (Als3), respectively, have demonstrated protection in mice against oropharyngeal and vaginal candidiasis and partial protection for systemic disease after intravenous challenge with *C. albicans* (18–20). The reduced protection against systemic candidiasis was correlated with the ability of *C. albicans* to regulate a variety of virulence factors in the host, allowing this species to adapt and evade the immune response in different niches (15, 16, 21). As suggested by Cassone (15), a multivalent vaccine formulation targeting two or more unrelated virulence factors has a better chance of preventing systemic candidiasis, reducing the probability of immune evasion, and increasing the antibody titer and strength of T-cell response. Accordingly, inactivated whole-cell formulations and fungal extracts also elicited a protective effect, but the presence of uncharacterized antigens and batch-to-batch inconsistencies impaired their investigation in clinical trials (16). In addition, live (attenuated) strains are not considered safe in immunocompromised/immunosuppressed patients.

The potential use of extracellular vesicles (EVs) as a multivalent vaccine formulation has emerged (22–26). EVs are nanosized particles surrounded by a lipid bilayer and carrying a diversity of structures, including native antigens of multiple nature, such as proteins, nucleic acids, lipids, and glycans (27). Our prior research demonstrated that fungal EVs released by yeasts of *C. albicans* activated murine macrophages, inducing the production of nitric oxide and the cytokines IL-12, TGF-β, and IL-10 (28). Similarly, treatment of murine bone marrow-derived dendritic cells (BMDCs) with *C. albicans* EVs regulated the production of IL-12p40, IL-10, and TNF-α, and induced a higher expression of major histocompatibility antigens (MHCII) and the co-stimulatory molecule CD86 (28). These results, associated with the ability of EVs to protect the insect *Galleria mellonella* against a lethal challenge with *C. albicans*, suggested that these compartments triggered the innate immune response and could act as a bridge between the innate and adaptive response (22, 29). Supporting this hypothesis, vaccination of mice with *C. albicans* EVs induced the production of specific IgG antibodies, modulated cytokines, and remarkably, fully protected immunosuppressed mice from lethal *C. albicans* infection (22).

The exact molecules carried by EVs and involved with the activation of phagocytes, key players during the innate immune response against fungal infections, were not identified. *C. albicans*, as well as other fungal species, displays a diversity of pathogen-associated molecular patterns (PAMPs), including mannoproteins, *β*-1,3-glucan, chitin, and glycolipids (30). These PAMPs, considered as molecular immunogenic signatures, are recognized by soluble and/or membrane-bound pattern recognition receptors (PRRs) (31). The PAMPs–PRRs interplay controls the innate immune response initiated by phagocytes. Fungal EVs lack the cell wall layer, but they can be decorated with mannoproteins and convey polysaccharides, such as *β*-1,3-glucan and glucuronoxylomannan (GXM) (32, 33).

In this study, we investigated the major PAMPs and PRRs involved with the engagement of EVs from *C. albicans* yeasts and murine BMDCs. We compared the stimulation of BMDCs using EVs from wild-type and knockout (KO) strains of *C. albicans* producing truncated mannoproteins and observed that the decoration of the EVs could specifically modulate which PRRs are activated. Furthermore, we identified that the protective effect mediated by vaccination with WT EVs is Toll-like receptor (TLR) dependent. Our results suggested that fungal EV compounds could be exploited in vaccine formulation to specifically activate one or more PRRs in phagocytes.

## RESULTS

### *C. albicans* EVs from strain ATCC 90028

**Fig 1:**
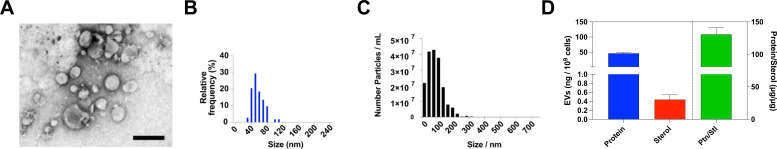
Properties and physical characterization of EVs released by *C. albicans* strain 90028. Transmission electron micrographs from negative contrasting EVs isolated from *C. albicans* strains (**A**). Size distribution was obtained by selecting random micrographs in which EVs were manually measured using ImageJ (**B**) and by NTA (**C**). Protein (**D**) and sterol (**D**) concentrations were measured in EVs. Results represent the average of three independent EV isolation experiments, and error bars represent the standard deviation. The protein:lipid ratio was calculated (**D**).

Isolated EVs were submitted to negative staining and were visualized by transmission electron microscopy (TEM), which revealed round and oval-shaped EVs, consistent with previous publications (Fig. 1A) (28, 34). Manual measurements of 150 EVs from 10 randomly selected TEM micrographs showed EVs with diameters ranging between 30 and 120 nm (Fig. 1B). The same EVs also had their dimensions determined by nanoparticle tracking analysis (NTA) and exhibited sizes ranging from 20 to 220 nm in diameter (Fig. 1D). Thus, distinct sizes of EV varied with the strain and method of purification employed, which underlines observations of previous studies (32). For this reason, we looked for other quantitative parameters, and for our subsequent experiments, we normalized the vesicle samples based on protein content. The total content of protein and sterol was determined for each EV batch, and the protein:lipid ratio was calculated for further comparison (Fig. 1D). As a control of contamination, aliquots of EV preparations were plated onto Sabouraud agar plates, with the absence of *Candida* growth after 72 h, confirming that the samples were sterile (data not shown).

### *C. albicans* EVs stimulate IL-6 production in a TLR4-dependent manner

**Fig 2:**
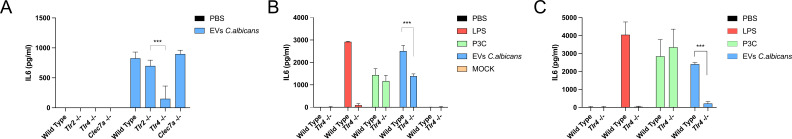
IL-6 production in response to *C. albicans* EVs is impaired in the absence of TLR4. (**A**) BMDCs derived from wild type (WT) and *Tlr2*^-/-^, *Tlr4*^-/-^, and *Clec7a*^-/-^ mice were stimulated with *C. albicans* EVs (strain 90028) for 24 h, and the levels of IL-6 were measured in the culture supernatants. MOCK-EVs (**B and C**) or treatment with PMXb (**C**) was also used to confirm that TLR4 activation is not mediated by LPS contaminants (B and C, respectively). LPS and P3C were used as positive controls. Error bars represent the standard deviation. Results represent the average of three independent experiments (*n* = 4). Statistical analysis was performed using two-way ANOVA and was analyzed by Tukey’s multiple comparisons test. ***P = 0.0007.

To identify the innate immune receptors potentially involved in the response to the *C. albicans* EVs, we first evaluated whether the absence of TLR2, TLR4, and dectin-1 would impact the production of IL-6 by BMDCs stimulated with *C. albicans* EVs (strain 90028). Confirming previous results, EVs from *C. albicans* yeasts were able to induce IL-6 production in wild-type (WT) cells (22). Deletion of TLR2 and dectin-1 did not impair IL-6 release; however, the absence of TLR4 significantly reduced the amount of IL-6 (Fig. 2A), suggesting that this PRR is one of the major receptors involved with *C. albicans* EV recognition by BMDCs. To exclude the possibility of LPS contamination, we performed two control experiments. First, a MOCK-EV preparation was developed, where all steps used to isolate EVs were performed from sterile culture medium. The fraction corresponding to the EVs was then incubated with BMDCs, and the IL-6 released was measured 24 h later. There was no IL-6 release under these conditions (Fig. 2). Second, the BMDCs were treated with polymyxin B (PMXb) for 30 min and then the stimuli (LPS or EVs) were added to the wells. After 24 h, the IL-6 levels were measured in the supernatant. Pre-treatment with PMXb abolished the effect of LPS, but the EVs’ ability to activate BMDCs and promote IL-6 release was not affected (Fig. 2).

### IL-10 and TNF-α are regularly produced by *Tlr4^-/-^* BMDCs

**Fig 3:**
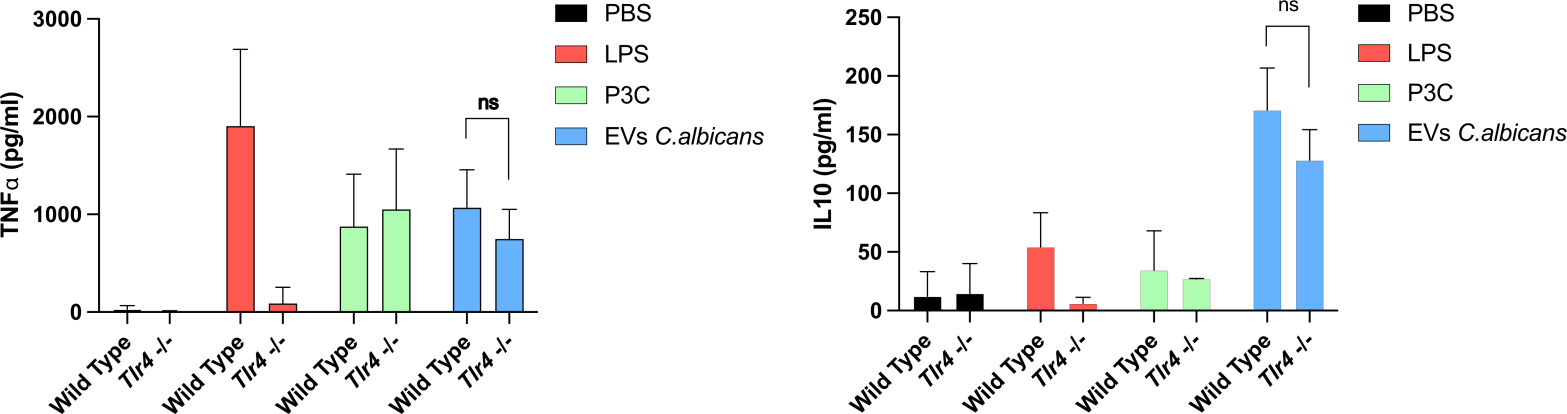
TNF-α, IL-10, and production after stimulation of BMDCs with EVs are not regulated by TLR4. WT and *Tlr4^-/-^* BMDCs were treated with *C. albicans* EVs (strain 90028), and the levels of IL-6, TNF-α, and IL-10 were measured in the culture supernatants by ELISA. Error bars represent the standard deviation. Results represent the average of three independent experiments (*n* = 4). Statistical analysis was performed using two-way ANOVA and was analyzed by Tukey’s multiple comparisons test.

IL-6 is a pleiotropic cytokine and fundamental during murine protection against systemic candidiasis caused by *C. albicans* (35). However, other cytokines may impact the immune response during *C. albicans* infection. Our results showed that although levels of IL-6 are reduced in *Tlr4^-/-^* BMDCs stimulated with *C. albicans* EVs when compared to the WT, the production of IL-10 and TNF-α appeared to be unaffected, suggesting that other PAMPs may participate during the recognition of EVs by BMDCs (Fig. 3).

### IL-6 production by THP-1 cells stimulated with *C. albicans* EVs is also mediated by TLR4

**Fig 4:**
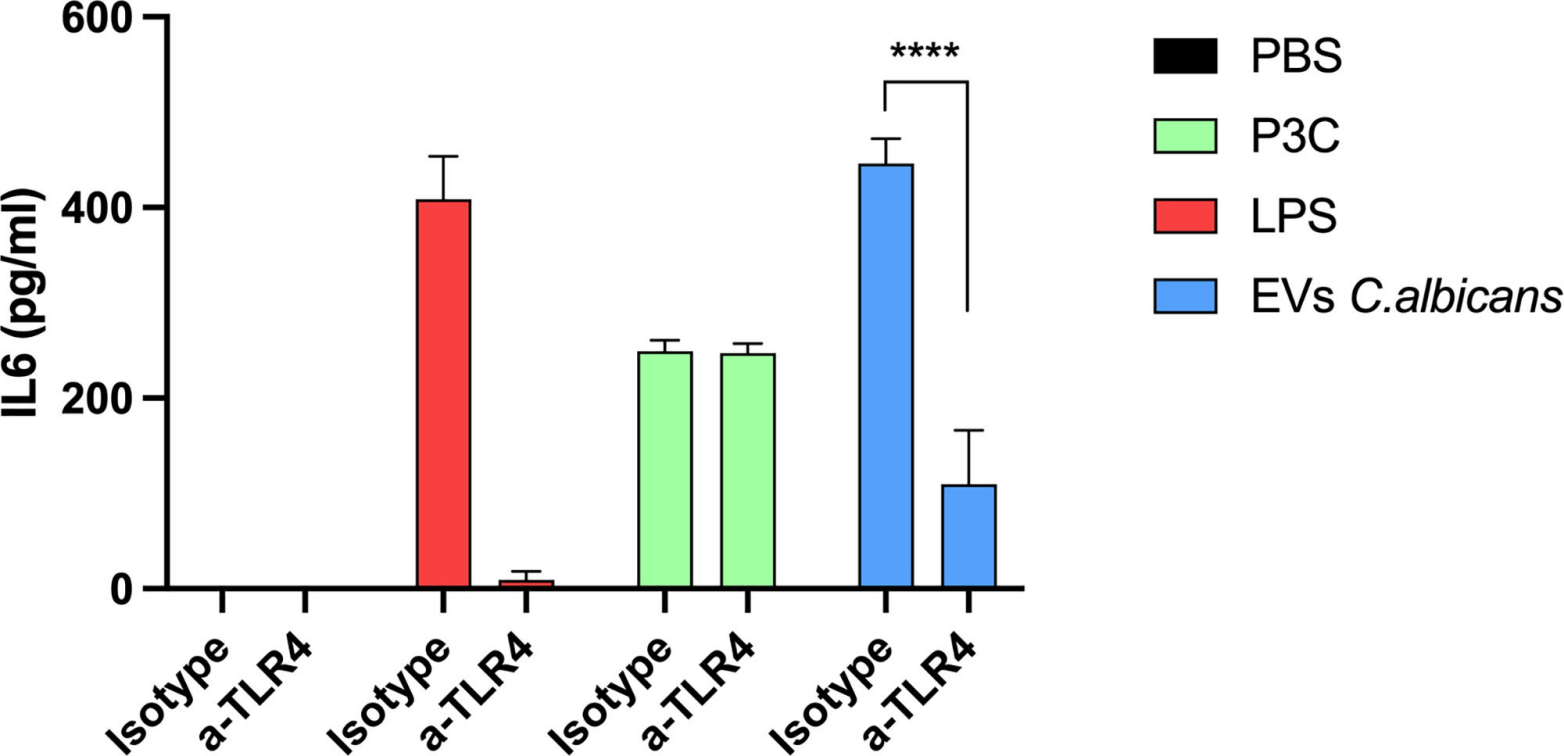
Antibody blockade of TLR4 hampers IL-6 production in human monocytes stimulated with *C. albicans* EVs. PMA-activated THP-1 cells, preincubated or not with anti-TLR4 antibodies, were stimulated with *C. albicans* EVs (strain 90028) for 18 h. IL-6 production was measured in the culture supernatants by ELISA. Error bars represent the standard deviation. Results represent the average of three independent experiments (*n* = 3). Statistical analysis was performed using two-way ANOVA and was analyzed by Tukey’s multiple comparisons test. ****P < 0.0001.

In order to confirm whether *C. albicans* EVs induce the production of IL-6 in human phagocytes, we used THP-1 cells activated with phorbol-12-myristate-13-acetate (PMA). Treatment of THP-1 cells with *C. albicans* EVs significantly enhanced IL-6 production (Fig. 4). Remarkably, the pre-treatment of THP-1 cells with anti-human TLR4, an antibody specifically used to block TLR4, reduced the production of IL-6 to basal levels, confirming that this PRR may act as a major receptor for *C. albicans* EVs also in human phagocytes.

### *C. albicans* EVs from parental strain NGY152 and mutants (*mnt1/mnt2*Δ, *mnn*Δ^6^, and *pmr1*Δ)

**Fig 5:**
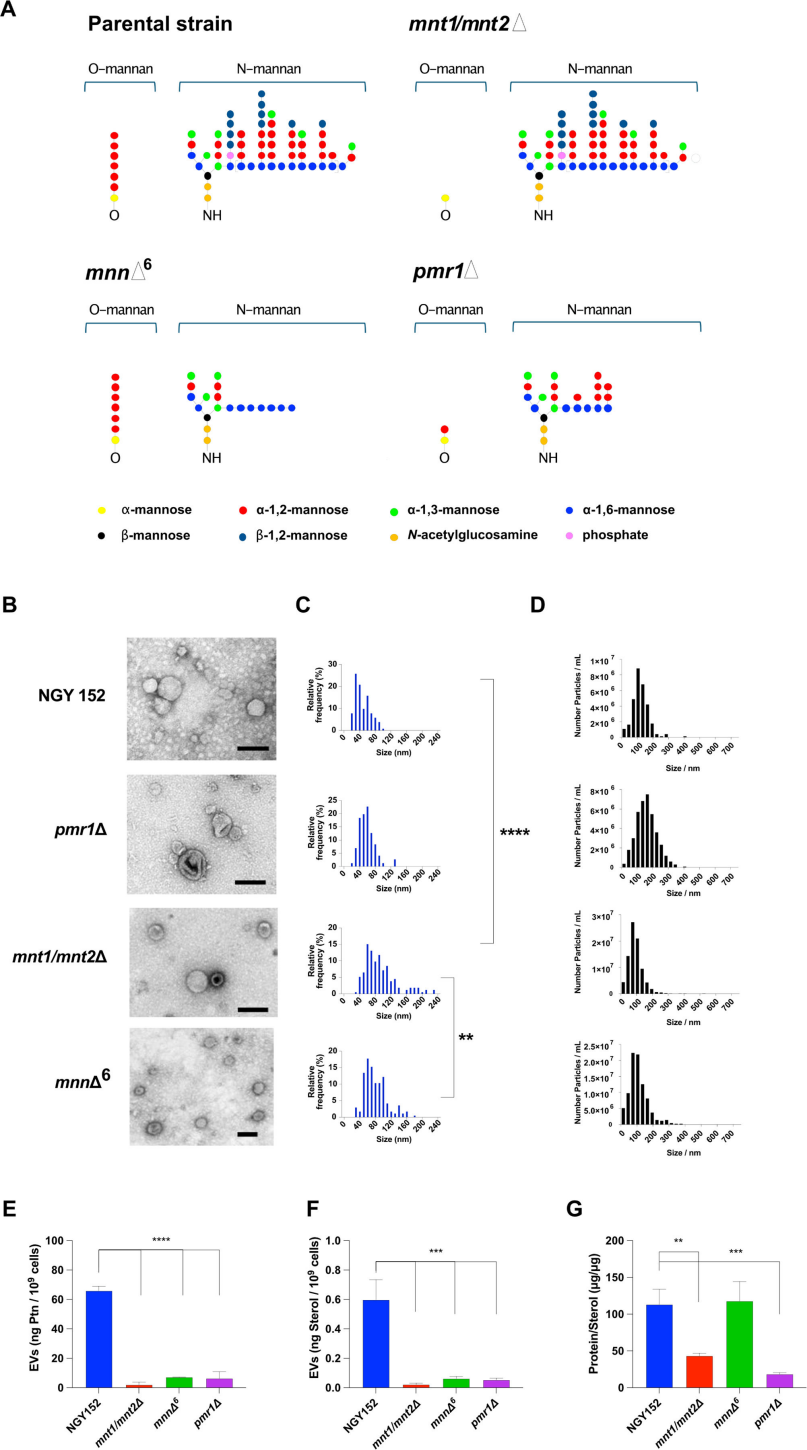
Properties and physical characterization of EVs released by *C. albicans* strain NGY152 and mutant strains (*mnt1/mnt2*Δ, *mnn*Δ^6^, and *pmr1*Δ). (**A**) Differences in *N*- and *O*-linked mannans found in the different strains are shown. (**B**) Transmission electron micrographs from negative contrasting EVs isolated from *C. albicans* strains. Size distribution was obtained by selecting random micrographs in which EVs were manually measured using ImageJ (**C**) and by NTA (**D**). Protein (**E**) and sterol (**F**) concentrations were measured in the different EVs. Results represent the average of three independent EV isolation experiments, and error bars represent the standard deviation. The protein:lipid ratio was calculated (**G**). Statistical analysis was performed using one-way ANOVA and was analyzed by Tukey’s multiple comparisons test. **P < 0.001 and ****P < 0.0001.

Although the physical-chemical properties of *C. albicans* EVs have been studied in detail (28, 34, 36, 37), it is still obscure whether specific mutations or particular strains produce EVs similar to those described for wild-type isolates. Therefore, we analyzed the properties of EVs produced by the parental and mutant strains used in the next experiments (NGY152, *mnt1/mnt2*Δ, *mnn*Δ^6^, and *pmr1*Δ). The latter represent mutants with alternations in mannosylation of the cell wall (38–40). For clarity, Figure 5A shows the defects in mannosylation of the cell wall displayed by each mutant strain. Manual measurements of TEM micrographs showed that EVs from the parental strain (NGY152) and the *pmr1*Δ displayed similar sizes when compared to EVs released by strain 90028, ranging between 30 and 120 nm (Fig. 5B). However, the strains *mnn*Δ^6^ and *mnt1/mnt2*Δ also displayed a population of larger EVs between 120–160 and 120–240 nm in diameter, respectively (Fig. 5C). As observed for EVs released by strain 90028, the NTA showed EVs ranging between 20 and 300 nm (Fig. 5D). As performed for the EVs from strain 90028, the total content of protein and sterol was determined for each EV batch, derived from the parental and mutant strains, and the protein:lipid ratio was calculated. When compared with EVs from the 90028, NGY152 and *mnn*Δ^6^ strains displayed similar proportions of protein/sterol. Strains from the *mnn*Δ^6^ and *mnt1/mnt2*Δ glycosylation mutants had a high sterol content (Fig. 5E through G). Based on the total content of proteins and sterol, EVs seem to be decreased in the mutants when compared to the parental strain.

### Mannoproteins and *β*-1,3-glucans carried by *C. albicans* EVs are responsible for the induction of IL-6 in murine BMDCs

Activation of TLR4 by *C. albicans* cells seems to be mediated by *O*-linked mannans in human monocytes and murine macrophages (41). Based on this finding, we investigated whether the TLR4 response induced by *C. albicans* EVs was elicited by the same compounds. EVs released by *C. albicans* strain NGY152 (parental strain) and three isogenic mutant strains, *mnt1/mnt2*Δ, *mnn*Δ^6^, and *pmr1*Δ, were used in these experiments. The mutant strain *mnt1/mnt2*Δ has the first ether-linked mannose but lacks the outer *O-*linked mannose sugars, whereas *mnn*Δ^6^ has marked deficiencies to synthesize the lateral branches of *N*-linked mannan (40). The *pmr1*Δ strain has a severe truncation in *O*-mannans and a partial alteration in *N*-linked mannan outer chain glycosylation (42). Phosphomannans are also markedly depleted in *mnn*Δ^6^ and *pmr1*Δ strains (40).

**Fig 6:**
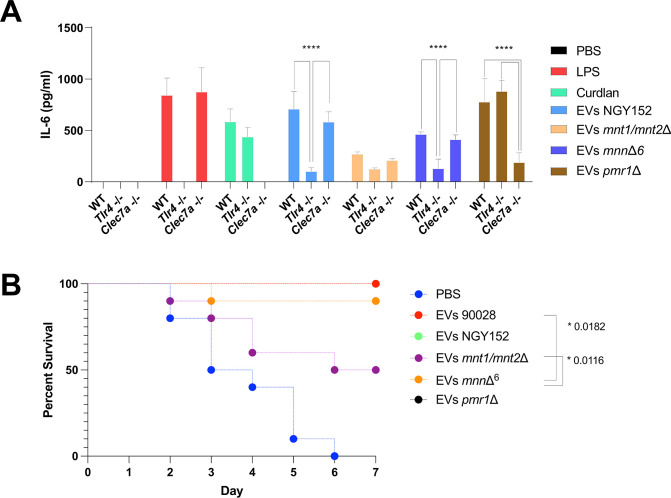
The impact of mannosyl residues carried by *C. albicans* EVs for IL-6 production by BMDCs and *G. mellonella* protection against lethal candidiasis. (**A**) WT, *Tlr4^-/-^*, and *Clec7a*^-/-^ BMDCs were treated with *C. albicans* EVs released by 90028, NGY152, *mnt1/mnt2*Δ, *mnn*Δ^6^, and *pmr1*Δ strains, and the levels of IL-6 were measured in the culture supernatants by ELISA. Error bars represent the standard deviation. Results represent the average of three independent experiments (*n* = 3). Statistical analysis was performed using two-way ANOVA and was analyzed by Tukey’s multiple comparisons test. ****P < 0.0001. (**B**) Insects were inoculated with EVs (10 µL of EV suspensions at 100 µg/mL) released by strains NGY152, *mnt1/mnt2*Δ, *mnn*Δ^6^, and *pmr1*Δ. Two days after EV inoculation, the insects were infected with 10 µL of a suspension containing 2 × 10^5^ yeasts of *C. albicans* (strain NGY152). Mortality was monitored for 7 d (*n* = 10). Statistical analysis was performed using one-way ANOVA, and the difference between groups was analyzed by log-rank (Mantel–Cox) test. *P* = *0.0182 and *0.0116.

IL-6 was produced by BMDCs treated with EVs from all KO strains, but significant differences in the cytokine’s levels were detected when the different EVs were compared (Fig. 6). As observed for the EVs from strain 90028 (Fig. 3 and 4), production of IL-6 by WT BMDCs stimulated with EVs from the parental strain (NGY152) was TLR4 dependent (Fig. 6A). A small and not significative difference was observed in the absence of dectin-1. Remarkably, IL-6 release was strongly reduced when BMDCs were stimulated with EVs from the *mnt1/mnt2*Δ strain, independently of the presence of *Tlr4^-/-^* or *Clec7a*^-/-^. These data suggest that the *O*-mannans are required to stimulate regular levels of IL-6 through TLR4 signaling. Corroborating with that, EVs from the strain specifically lacking the *N*-glycans (*mnn*Δ^6^ strain) displayed a similar ability to stimulate IL-6 when compared with EVs from 90028 and the parental strains. These data indicate that when *O*-mannans are carried by EVs, TLR4 seems to be the major receptor involved with IL-6 synthesis (Fig. 6A). In contrast, TLR4 does not appear to be necessary for activation of BMDCs by EVs from *pmr1*Δ. However, IL-6 production by *Clec7a*^-/-^ cells stimulated with these EVs was significantly reduced. Together, these results strongly suggest that *O*-linked mannans engage with TLR4 in murine BMDCs and indicated that a dectin-1 ligand, possibly *β*-1,3-glucans, is also carried by *C. albicans* EVs. In control experiments, LPS and curdlan had no stimulatory effect on *Tlr4^-/-^* and *Clec7a*^-/-^ cells, respectively (Fig. 6A).

All EVs, independent of the strain, were able to promote IL-6 production by BMDCs. We, therefore, also compared their ability to protect larvae from *G. mellonella* against a lethal dose of *C. albicans*. Except EVs from strain *mnt1/mnt2*Δ that induced partial protection, all other EVs elicited a strong and protective effect against a subsequent challenge with yeasts of *C. albicans*, suggesting that other PAMPs could stimulate the protective response and that fungal EVs could be further exploited as activators of specific PRRs (Fig. 6B).

We then investigated whether two major cell wall components, chitin oligomers and *β*-1,3-glucans, are carried by *C. albicans* EVs. Using wheat germ agglutinin (WGA) and dectin-1-Fc (IgG) fusion proteins, we confirmed that both chitin-related structures and *β*-1,3-glucans were EV associated. As shown in Table 1, the EVs from NGY152 and 90028 strains displayed similar contents of chitin oligomers and *β*-1,3-glucans. EVs from the *mnt1/mnt2*Δ and *mnn*Δ^6^ strains displayed a small decrease in *β*-1,3-glucans when compared to the parental strain. However, we observed a substantial increase in chitin oligomer content in EVs released by the *mnt1/mnt2*Δ strain. EVs from the *pmr1*Δ strain showed a higher content of *β*-1,3-glucan, possibly impacting the BMDC recognition.

**TABLE 1:** Chitin and *β*-1,3-glucan content in *C. albicans* EVs^*a*^

Strain	Chitin-related structures (ng/μg of ptn)	*β*-Glucan (ng/μg of ptn)
ATCC 90028	62.0 ± 4	61.6 ± 7
NGY152 (parental)	56.7 ± 6	68.5 ± 2
NGY 112 (*mnt1/mnt2*Δ)	68.9 ± 4	61.1 ± 2
NGY 600 (*mnn*Δ^6^)	60.4 ± 2	59.4 ± 2
NGY 355 (*pmr1*Δ)	68.7 ± 3	81.3 ± 3

^*a*^ Means ± SD (*n* = 3) are given.

### Zeta potential (ζ) is modified in *C. albicans* EVs lacking *N-* and *O-*linked mannans

As the binding of nanoparticles to cell membranes is influenced by their surface charge, we investigated the zeta potential of EVs from *C. albicans*. Similar results were obtained for the EVs released by the WT, parental, and *mnn*Δ^6^ strains with values of –24.53, –25.15, and −28.45 mV, respectively (Table 2). Remarkably, the EVs produced by strains *mnt1/mnt2*Δ and *pmr1*Δ displayed significant and contrasting changes. The lack of the *O-*linked mannan resulted in an increased value of −18.97 mV, whereas the truncation of *N*- and *O-*linked mannan (*pmr1*Δ) resulted in a reduction of the zeta potential to −48.61 mV for the EVs.

**TABLE 2:** Zeta potential of *C. albicans* EVs^*a*^

Strain	Zeta potential (mV)	SD
ATCC 90028	–24.53	±3.54
NGY152 (parental)	–25.15	±4.97
NGY 355 (*pmr1*Δ)	–48.61	±6.29
NGY 112 (*mnt1/mnt2*Δ)	–18.97	±2.99
NGY 600 (*mnn*Δ^6^)	–28.45	±2.66

^*a*^ Means ± SD (*n* = 3) are given.

### The absence of TLR4 leads to loss of protection in a murine model of systemic candidiasis

**Fig 7:**
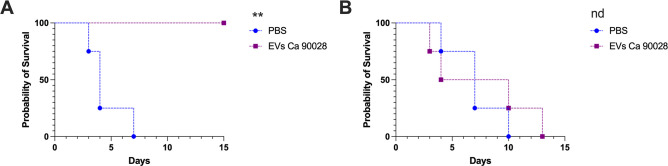
Protection induced by *C. albicans* EV vaccination in a systemic murine model of candidiasis is TLR4 dependent. Wild type (**A**) and TLR4 KO (**B**) mice were immunized with *C. albicans* EVs (strain 90028), immunosuppressed with CP, and then challenged with a lethal inoculum of *C. albicans*. Statistical analysis was performed using one-way ANOVA, and the difference between groups was analyzed by log-rank (Mantel–Cox) test, **P = 0.0069. Results are representative of two independent experiments (*n* = 7).

We recently demonstrated that vaccination of Balb/c mice with *C. albicans* EVs fully protected these animals in a lethal candidiasis model (22). Here, we investigated the protective effect of *C. albicans* EVs in C57BL/6 mice and evaluated whether the protection was TLR4 dependent using *Tlr4^-/-^* mice. Our results showed that immunization of WT C57BL/6 mice with *C. albicans* (strain 90028) EVs was effective (Fig. 7A), confirming that vaccination worked independent of the mice background. All non-vaccinated mice exhibited declining health and were sacrificed up to 8 d after infection (Fig. 7A), whereas vaccination promoted full protection (Fig. 7A). Corroborating with our previous experiments using phagocytes and EVs from *C. albicans* strain 90028, the protective effect was lost in *Tlr4^-/-^* mice (Fig. 7B). Of note, *Tlr4^-/-^* mice were apparently more resistant to *C. albicans* infection, because these animals survived modestly longer than the WT mice. However, vaccination of *Tlr4^-/-^* mice with EVs had no impact on mice survival (Fig. 7B).

### The protective effect mediated by EVs from *C. albicans* is not strain specific

**Fig 8:**
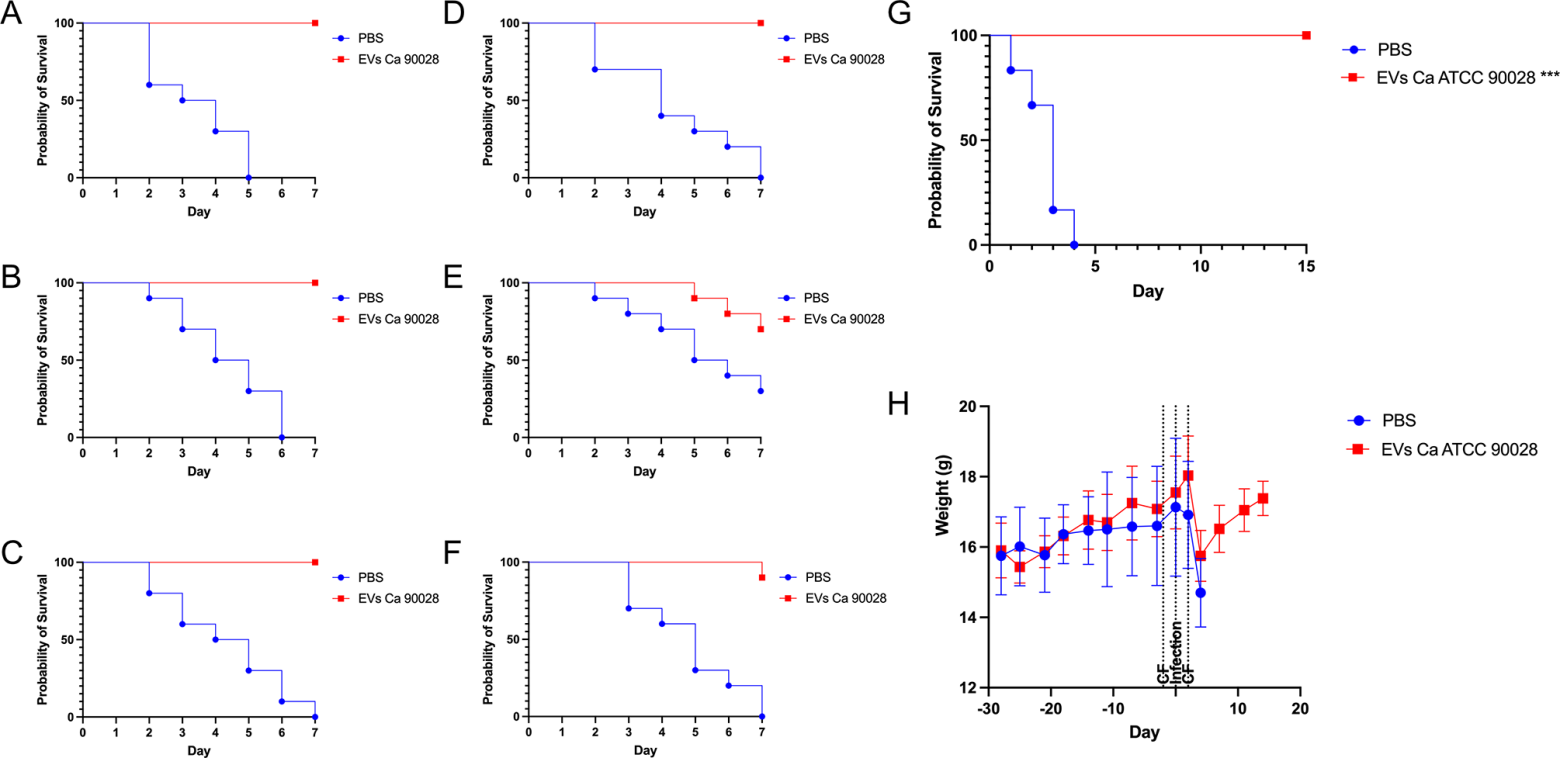
EVs produced by *C. albicans* strain 90028 protect animals against other strains. Insects were inoculated with EVs (10 µL of EV suspensions at 100 µg/mL) released by strain 90028 (**A–F**). Two days after EV inoculation, the insects were infected with 10 µL of a suspension containing 2 × 10^5^ yeasts of *C. albicans* clinical strains (**A**) OD2, (**B**) OD6, (**C**) OD7, (**D**) OD9, and (**E**) OD10 or the (**F**) ATCC strain SC5314. Mortality was monitored for 7 d (*n* = 10). (**G**) Survival rate and (**H**) weight (mean ± SD) of the mice immunized with *C. albicans* EVs (strain 90028), immunosuppressed with CP, and then challenged with a lethal inoculum of *C. albicans* ATCC SC5314. Statistical analysis was performed by log-rank (Mantel–Cox) test. *P = 0.0001 and **P = 0.0007.

To confirm that the protective effects were not strain specific, two additional experiments were conducted. First, larvae from *G. mellonella* were immunized with EVs from *C. albicans* strain 90028 and then infected with distinct *C. albicans* strains, including the ATCC SC5314 strain and five clinical isolates (Fig. 8A through F). Regardless of the strain used for infection, the pre-treatment with EVs resulted in complete protection, whereas non-treated larvae succumbed to the infection within 6 to 7 d. Second, the protective capacity of EVs from strain 90028 was tested in mice against a lethal challenge using the *C. albicans* strain SC5314. Following the same vaccination and immunosuppression protocol described in this section, all mice vaccinated with EVs survived up to 15 d after infection, whereas phosphate-buffered saline (PBS)-treated mice died within 3 d (Fig. 8G). Mice weight was also accompanied (Fig. 8H). To assess the presence of *C. albicans* in the protected animals, euthanasia was performed, and fungal burden was evaluated in the kidney, liver, and spleen. After 48 h of culture, no colonies were detected, indicating that the vaccination not only shielded the mice from infection but also resulted in organ sterilization (data not shown).

## DISCUSSION

In previous studies, we demonstrated that fungal EVs activated BMDCs and were successfully used as vaccine formulation to prevent systemic candidiasis in immunosuppressed mice (22, 28, 34). However, the molecules and mechanisms triggering the protective response were not examined. Using specific probes and *C. albicans* strains with defects in mannosylation, we confirmed that *O*-linked mannans and *β*-1,3-glucans are PAMPs carried by EVs that activate BMDCs. In addition, for our prototype vaccine formulation, in which only EVs from *C. albicans* strain 90028 were used, TLR4 was required for murine protection.

The combination of EV measurements confirmed that all strains released EVs ranging from 30 to 300 nm in diameter, consistent with EVs produced by fungi (43–47). However, a higher frequency of smaller particles was observed by TEM for all strains, suggesting the limitations of using a single method for EV size determination. The International Society for Extracellular Vesicles (ISEV) recently asserted that as yet, there is no standard method for EV quantification and characterization, the protein content and the total particle number are the most commonly adopted methods for making comparisons of EVs (27). Taking that our size measurements showed different values for fungal EVs, we decided to use the protein content to normalize our samples and perform the functional studies. The differences observed in the protein/sterol ratio is indicative that the presence of mannan chains may be associated with protein cargo addressing. However, further studies are needed to confirm this hypothesis.

Earlier proteomic analyses of *C. albicans* EVs confirmed the presence of several glycoproteins, such as MP65, Als4, chitinases 2 and 3, and PR1a, among others (28, 34, 36). Consistent with that, we also demonstrated previously that proteins carried by *C. albicans* EVs were recognized by ConA, a lectin used as a probe to recognize *α*-D-mannosides (28). We then started our functional studies testing whether the activation mediated by *C. albicans* EVs would involve TLR2 and TLR4, some of the major PRRs that recognize fungal mannoproteins (41, 48). In addition, as Higuchi and colleagues demonstrated that EVs from *Saccharomyces cerevisiae* carry small amounts of *β*-1,3-glucan (33), the influence of dectin-1 in our model was also investigated. Although the IL-6 production by BMDCs treated with EVs from the *C. albicans* strain 90028 was TLR4 dependent, IL-10 and TNF-α were produced at similar levels when WT and *Tlr4^-/-^* BMDCs were stimulated with fungal EVs. These results suggested that TLR4 was possibly a major receptor involved with the BMDC activation, but other PAMPs carried by *C. albicans* EVs could modulate phagocytes. The presence of an outer layer of mannoproteins in fungal EVs was demonstrated recently by Rizzo and colleagues (32). Their study showed that a large population of EVs from *Cryptococcus neoformans*, *S. cerevisiae*, and *C. albicans* depicted an external layer of fibrils. The TEM images of fungal EVs resembled the outer layer of cell walls from *C. albicans* and *S. cerevisiae*. In addition, the EVs released by *C. neoformans* were ConA reactive, suggesting the presence of mannosylated structures (32). TLR4 dependence was confirmed using *C. albicans* EVs and THP-1 cells, a human monocyte cell line, confirming the TLR4 requirement for an effective response.

The mannoprotein layer of *C. albicans* cell wall consists of an *N-* and *O*-linked mannans network with a spatial distribution that directly impacts the host cell activation (31, 41, 49). Using a combination of *C. albicans* isogenic mutants with defects in glycosylation, Netea and colleagues elegantly showed a correlation between the thickness and composition of the cell wall and its effect on PRR activation (41). They demonstrated that fungal recognition by human monocytes and murine macrophages can occur through multiple systems, including the mannose receptor (MR), TLR4, and the collaborative work of TLR2 and dectin-1 (31). In addition, they found that linear *O*-linked mannans were specifically recognized by TLR4. We then speculated whether the same group of glycans could be carried by *C. albicans* EVs and activate murine BMDCs. To investigate this hypothesis, we compared the modulatory effect of EVs released by a combination of isogenic mutant strains of *C. albicans* lacking the ability to build regular mannoproteins. Mnt1p and Mnt2p are partially redundant *a*-1,2 mannosyltransferases involved in *O*-glycolysation (39). Deletion of both proteins culminates with truncated *O*-mannans. The *mnn*Δ^6^ strain displays *O*-linked mannans and the *N*-linked *α*-1,6-mannan core, but lacks the ability to produce regular branched *N*-linked mannans (42). An increase in N-acetylglucosamine and glucose content was observed in the cell wall of this mutant, supported by a higher reactivity with Fc-dectin-1 and WGA (42). The absence of Pmr1p (*pmr1*Δ strain) reduces the magnesium availability in the Golgi, compromising the activity of glycosyltransferases and promoting an impact in both *N-* and *O*-linked mannans (38). The *N-*linked lateral branch was reduced and the phosphomannan chain was absent, whereas the *O*-linked mannan was shorter, lacking Man_3_-Man_5_.

Considering that ATCC 90028 and the parental strain (NGY152) share normal *N-* and *O-*glycosylation, it becomes evident that, under typical conditions (such as with WT strains), TLR4 emerges as the principal receptor engaged by *C. albicans* EVs. However, we acknowledge the possibility that the distribution of ligands on EV surfaces might vary depending on the specific strain being studied. Thus, it is reasonable to anticipate that distinct strains of *C. albicans* might release EVs with varying capacities to activate phagocytes.

The hypothesis implicating *O*-linked mannans in TLR4 activation was supported by the fact that lower levels of IL-6 were produced by BMDCs stimulated with EVs from the *mnt1/mnt2*Δ strain, whereas the EVs produced by the *mmn*Δ^6^ mutant, bearing normal chains of *O*-linked mannans, behaved similarly to the 90028 and parental strains. Therefore, TLR4-dependent responses seem to occur when *O*-linked mannans are present. In the *C. albicans* cell wall, mannoproteins can reduce or completely inhibit the exposure of *β*-1,3-glucans, thereby blocking dectin-1 access. However, in mutant strains with reduced mannoprotein levels, dectin-1 engagement and subsequent cytokine production may occur (50). Our results suggest a comparable spatial distribution of *β*-1,3-glucans and mannoproteins in EVs released by *C. albicans.* This hypothesis was reinforced by the experiments performed using EVs produced by the *pmr1*Δ strain, in which the synthesis of *N-* and *O*-linked mannans is impaired. Unlike the *mnt1/mnt2*Δ strain, these EVs promoted higher levels of IL-6 production, confirming that BMDCs can be activated even in the absence of typical *O*-linked glycans, but only whether the *N*-linked glycans are absent or truncated. Based on these findings, it is conceivable that in the absence of both *N-* and *O*-linked glycans, exposure to other ligands may occur. Because IL-6 production was significantly reduced in *Clec7a*^-/-^ BMDCs treated with EVs from this strain, the response appears to be dectin-1 dependent.

So far, the presence of structural polysaccharides was never investigated in EVs from *C. albicans*, but several enzymes related to carbohydrate metabolism have been characterized as EV components in proteomic analysis (29, 34, 36). In addition, fungal EVs were linked with cell wall construction in *Aspergillus fumigatus*, carrying a mixture of chitin and glucan synthases (45). Together, these data suggest a close association between fungal EVs and these structural polysaccharides. Indeed, the presence of chitin-related structures and *β*-1,3 glucans in *C. albicans* EVs could contribute to BMDC activation. For example, the presence of chitin oligomers could be associated with the IL-6 and IL-10 release (51–53), even in the absence of TLR4. Because the *β*-1,3-glucan levels are similar in EVs from both 90028 and NGY152, a possible explanation for our results would be a higher access of dectin-1 to *β*-1,3-glucan at the surface of EVs from NGY152 and specially in the EVs from *pmr1*Δ strain. We cannot rule out that the higher content of *β*-1,3-glucan carried by EVs from the *pmr1*Δ strain can also have a major effect in BMDC activation.

Surface charges may play a crucial role in the stability of EVs and how they interact with host cells. Despite lacking phosphomannans, the strain *mnn*Δ^6^ displayed similar zeta potential values when compared to the 90028 and parental strain. This result suggested that the absence of phosphomannan is probably not linked to the reduced zeta potential of EVs from the *pmr1*Δ strain. Because we did not detect any disparity in the chitin oligomer levels when the *mnt1/mnt2*Δ and *pmr1*Δ were analyzed, and the *β*-1,3-glucans do not seem to impact charge reduction, we believe that *pmr1*Δ pleiotropic phenotype could be linked with a distinct net charge impacting the zeta potential. Taking that all EVs were able to modulate the phagocytes activity, it seems that the zeta potential changes do not impact the host cell activation. Additional experiments should be performed to investigate these differences in the EV charge.

The most efficient antifungal vaccines induce both cellular and humoral responses (16, 54, 55). Single antigens are usually formulated with adjuvants, regulating co-stimulatory molecules and cytokine production (56). However, the current adjuvants display significant drawbacks, which can compromise vaccine tolerability or safety (56). Consequently, the search for safe and nontoxic adjuvants is a major challenge. The use of natural and synthetic TLR4 agonists in vaccine formulations has been extensively tested in cancer and infectious disease vaccines (57). These agonists are known to stimulate both the innate and adaptive immune responses (58). For instance, the monophosphoryl lipid A (MPLA) was successfully used in human vaccine formulations against some viral infections (59). The *in vitro* mechanism triggered by the addition of MPLA included an increase in IL-12 production and a higher expression of MHCII and co-stimulatory molecules by BMDCs. TLR4 agonists also impact antibody production. For instance, Przetak and colleagues demonstrated that synthetic LPS analogs boost systemic and mucosal antibodies in mice when administered in combination with tetanus toxoid antigen (60). In addition, the use of E6020 (a nontoxic lipid A analog) during immunization with hapten 4-hydroxy-3-nitrophenyl acetyl was associated with increased and sustained antibody titers and promoted isotype class switching (61). As previously shown by our group, fungal EVs have similar *in vitro* effects and promoted full protection against candidiasis regardless of the presence of an adjuvant (22). Furthermore, the cytokine profile found in the spleen of infected animals demonstrated that immunization with EVs alone resulted in a lower inflammatory response when compared with EVs formulated with adjuvants, suggesting that the use of EVs could accelerate the recovery of the inflammatory phase. Finally, the *in vivo* experiments performed in this study demonstrated that the protective response elicited by vaccination with *C. albicans* EVs released by strain 90028 was mediated by a TLR4-dependent activation.

The combination of results presented here reinforced the application of fungal EVs as a multi-antigenic delivery system to prevent candidiasis and potentially other fungal infections. A clear advantage of using *C. albicans* EVs in vaccine formulation, besides the multitude of targets and safety to immunocompromised, is the presence of native TLR4 agonists, circumventing the requirement of synthetic adjuvants. According to our results, the PAMPs carried by *C. albicans* include mannoproteins and the polysaccharides *β*-1,3-glucans and chitin-derived structures. However, based on our findings, the PRRs involved will be dependent on the EVs surface, which seems to be strain dependent, where the polysaccharides could be sterically covered by a mannoprotein layer. At the same time, a combination of EVs could surpass the lack of specific PRRs and make the formulation useful in cases of PRR polymorphisms. These results indicated that multiple combinations of fungal EVs could be tested to address specific innate immune receptors, opening new venues for fungal EVs in vaccination.

## MATERIALS AND METHODS

### Culture of fungal cells

*C. albicans* strains ATCC 90028, SC5314, NGY152 (parental strain) (62), NGY112 (*mnt1/mnt2*Δ), NGY600 (*mnn*Δ^6^) (42), and NGY355 (*pmr1*Δ) (38), and clinical strains OD2, OD6, OD7, OD9, and OD10 obtained from INCQS (Instituto Nacional de Controle de Qualidade em Saúde, Fiocruz, Brazil) were stored in Sabouraud broth [2% glucose (Sigma, USA) and 1% peptone (Acumedia, Brazil) with 15% glycerol (Merck, USA)] and were maintained at −80°C. Yeast cells were cultured in Sabouraud broth for 48 h at 30°C under agitation (150 rpm). This pre-inoculum was then transferred to an Erlenmeyer flask containing 1 L of liquid Sabouraud broth and was cultured under the same conditions.

### Preparation of fungal EVs

EV isolation was performed as established in our laboratory (37). Briefly, cultures were centrifuged at 4,000 × *g* for 15 min at 4°C. The supernatants were collected and further centrifuged at 15,000 × *g* for 15 min at 4°C to remove cell debris. Residual cells and debris were removed after a filtration step using a 0.45-µm membrane filter (Merck Millipore, USA). The cell-free supernatant was concentrated about 50 times using an Amicon ultrafiltration system (100-kDa membrane, Merck Millipore, USA). The concentrated supernatant (20 mL) was then ultracentrifuged at 100,000 × *g* for 1 h at 4°C in Beckman Optima LE-80K (70Ti Fixed-Angle titanium rotor). The pellet resultant was washed twice with PBS pH 7.4, at 100,000 × *g* for 1  h at 4°C. Fungal EVs were then suspended in PBS, and aliquots were plated onto brain heart infusion (BHI) agar (Sigma, USA) plates and were incubated for 72 h to confirm the absence of any contaminant, confirmed by no colony observation. Quantification of EVs was carried out using the quantitative Amplex Red Sterol Assay Kit (Invitrogen, USA) and BCA Protein Assay Kit (Thermo Fisher Scientific, USA).

### Nanoparticle tracking analysis

EV concentration and size distribution were measured using ZetaView nanoparticle tracking analyzer (NTA; Particle Metrix GmbH). EV suspensions were diluted in previously filtrated PBS (0.22 µm) for optimal concentration range. Software parameters were the temperature at 23°C, the sensitivity of 30–85 frames per second (fps), a shutter speed of 55, and a laser pulse duration equal to that of shutter duration. Acquisition parameters were set to a minimum brightness of 20, a maximum size of 200 pixels, and a minimum size of 5 pixels. Polystyrene particles (Microtrac GmbH) with an average size of 100 nm were used to calibrate the instrument. Data were analyzed using ZetaView software and GraphPad Prism 8 (GraphPad, California, USA).

### Electron microscopy

Vesicles morphology and size were determined by TEM using the negative staining technique. Briefly, 5 µL of previously purified EVs was adsorbed for 30 s onto a copper grid (300 mesh) coated with Formvar (polyvinyl resin, Ted Pella, Inc.). The excess was removed with a filter paper. Subsequently, the samples were contrasted with 2.5% uranyl acetate for 30 s and were placed in a vacuum desiccator until visualization. EVs were visualized in a transmission electron microscope (FEI Tecnai Spirit) operated at 120 kV. ImageJ software was used to measure the diameter of up to 100 EVs per sample.

### Cytokine production by BMDCs and THP-1 cells

BMDCs were generated as described previously (63), with modifications. Briefly, bone marrow was harvested from the tibia and femur of wild type, *Tlr2*^-/-^, *Tlr4^-/-^*, and *Clec7a*^-/-^ C57BL/6 mice. The cells were suspended at 10^6^ cells/mL in 10 mL RPMI 1640 (Gibco, USA), supplemented with 10% FBS (Gibco, Brazil), 1% Pen/Strep (Gibco, USA), and 20 ng/mL mouse rGM-CSF (Peprotech, USA) at 37°C, 5% CO_2_. After 3 d, 10 mL of fresh medium containing mouse rGM-CSF was added. The cells were collected by centrifugation at 200 × *g* for 10 min. BMDCs were plated in 96-well at a density of 2 × 10^5^ cells/well and were incubated for 18 h with *C. albicans* EVs (100 µg/mL, based on the protein content), after which the supernatant was recovered for cytokine determination. The concentrations of TNF-α, IL-6, and IL-10 were determined using enzyme-linked immunosorbent assay (ELISA). Measurements were performed in triplicate following the manufacturer’s instructions (BD Biosciences, USA). The MOCK-EVs group consisted of processing the Sabouraud medium replicating all steps for EV isolation, in the absence of *C. albicans*. Final suspension used in the experiment was normalized by the volume used in the group containing the EVs. Curdlan, LPS, and Pam3Cys were used as agonists for dectin-1, TLR4, and TLR2, respectively. For the polymyxin B-treated cells control, the suspension of BMDCs (2 × 10^5^ cells) was pre-incubated with 20 µg/mL of Polymyxin B (Sigma, USA) for 30 min at 37°C, 5% CO_2_. The cells were then incubated with EVs (100 µg/mL, based on the protein content) or 100 ng/mL LPS (Sigma, USA) and incubated for 18 h. PBS-treated cells were used as a control. After incubation, the supernatants were collected, and the cytokines were measured as described.

THP-1 cells (10^6^ /mL) were differentiated using 10 ng/mL phorbol 12-myristate 13-acetate (PMA; Sigma, USA) for 2 d. After removing the PMA containing media, the cells were incubated in RPMI (Gibco, USA) supplemented with 10% FBS and 1% Pen/Strep for 1 d and were used for subsequent treatments. THP-1 cells were incubated with neutralizing TLR4 (HTA 125) mAb (2 µg/mL) or isotype mAb (IgG – sc2025) (Santa Cruz Biotechnology, USA) for 40 min. Antibody-treated cells were incubated with LPS (100 ng/mL) or EVs (100 µg/mL, based on the protein content) for 18 h. Culture media were collected for IL-6 measurement ELISA MAX Standard Set (BioLegend, USA) following the manufacturer’s instructions.

### *β*-Glucan and chitin-related structures

The presence and quantification of *β*-glucan and chitin-related structures were performed using WGA- and dectin-1-Fc (IgG) fusion proteins using an inhibition ELISA (64). Briefly, a 96-well polystyrene microplate (Coastar, USA), reaction plate, was coated with 50 µL of chitin oligomers (Sigma, USA) or laminarin (Sigma, USA) diluted to 10 µg/mL in PBS and was incubated overnight at 4°C. The reaction plates were washed three times with PBS and were blocked with 1% BSA (Sigma, USA) for 1 h at 37°C. A second 96-well polystyrene microplate, inhibition plate, was also blocked for 1 h at 37°C with the same blocking buffer. After the blocking step, the ELISA plates were washed three times with PBS. For quantifications, a standard curve of chitin oligomers (prepared at 1 mg/mL) or laminarin (prepared at 10 mg/mL in PBS) was made with concentrations ranging from 100 µg/mL to 15.25 ng/mL (obtained by serial dilutions 1:2), and 50 µL of each concentration was added to the inhibition plate in duplicate. Still on the inhibition plate, EVs were plated following the same 1:2 dilution as the standard curve. Fifty microliters of a 4 µg/mL solution of WGA-Fc or dectin-1-Fc chimeras was added to all wells in the inhibition plate, respectively, containing chitin oligomers or laminarin, and then was incubated for 2 h at 37°C. The contents of the inhibition plate were then transferred to the respective wells in the reaction plate. Excess chimeras for chitin oligomers or laminarin were left to interact with the chitin and laminarin of the reaction plate, respectively, for 1 h at 37°C. The wells were then washed three times and were incubated with 50 µL of 1 µg/mL solution of HRO-conjugated anti-mouse IgG (Southern Biotech, USA) for 1 h at 37°C. The plates were then washed three times with PBS and were incubated with 50 µL of 3,3′, 5,5′-tetramethylbenzidine (TMB; Thermo Fisher Scientific, USA). The reaction was interrupted with 12.5 µL of 1 N HCl (Merck, USA), and the reading was performed in a microplate reader with a 450-nm filter (Biotek ELx808).

### *G. mellonella* infection

*G. mellonella* (final instar larval stage) was selected according to weight (0.25–0.30 g). Larvae (10 per group) were inoculated with 10 µL of EV suspensions (100 µg/mL per insect, based on the protein content) using a Hamilton syringe into the hemocoel through the last proleg with the EVs of the strain ATCC 90028, NGY152, *mnn*Δ^6^, and *pmr1*Δ. PBS was used as a negative control. The larvae were then placed in sterile Petri dishes and were kept in the dark at 37°C for 2 d. Subsequently, all larvae were inoculated with 10 µL of a suspension containing 2 × 10^5^ yeasts of *C. albicans* strains. Mortality was monitored by checking twice daily. Death was assessed by the lack of movement in response to stimulation. Survival curves were plotted, and statistical analyses were performed using the log-rank (Mantel–Cox) survival test, and displayed results represent the mean percentage survival of larvae from all assays.

### Mouse survival model

Female C57BL/6 wild type and *Tlr4*^-/-^ (6–8 wk, seven animals per group) were immunized intraperitoneally (i.p.) four times, 1 wk apart, with 200 µL of an EV suspension at a final protein concentration of 100 µg/mL in PBS. Four days after the third boost, the animals were immunosuppressed with 200 mg/kg of cyclophosphamide (CP) in PBS (Sigma, USA) intraperitoneally (65). Three days later, the animals were inoculated i.p. with a lethal inoculum of *C. albicans* ATCC 90028 or SC5314 (3 × 10^7^ yeasts per mouse); 2 d after infection, the animals were immunosuppressed again under the same conditions. Deaths were evaluated daily. Mice were treated according to the ethical guidelines for animal experimentation of the Federal University of Rio de Janeiro (Protocol 089/18).

### Zeta potential analysis

Zeta potential analysis of fungal EVs was recorded in triplicates in a final protein concentration of 100 µg/mL in PBS, pH 7.4. Zeta potential was determined by using a Zetasizer NanoZS90 (Malvern Instruments, Worcestershire, UK). Each zeta measurement was repeated three times. Statistical analysis was performed using one-way analysis of variance (ANOVA) and was analyzed by Dunnett’s multiple comparisons test. **P = 0.0089; ****P < 0.0001.

### Statistics

For statistical analyses, one-way ANOVA was used and analyzed by Dunnett’s multiple comparisons test, and two-way ANOVA was used and analyzed by Tukey’s multiple comparisons test, with a confidence interval of 95% and *P* < 0.05 considered statistically significant. For survival rates, statistical analysis was carried out and the difference between groups was analyzed by log-rank (Mantel–Cox) test; both were performed with the GraphPad 8 software.
